# High Treatment Burden and Low Quality of Life in Acute Kidney Injury Survivors: How Should We Respond?

**DOI:** 10.34067/KID.0000000713

**Published:** 2025-05-29

**Authors:** Alvin H. Moss

**Affiliations:** West Virginia University School of Medicine, Morgantown, West Virginia

**Keywords:** AKI

Patients with CKD are known to have a high symptom burden, one comparable with patients with cancer.^1^ Multiple studies have found that quality of life for patients with kidney disease is inversely related to symptom burden.^2,3^ The higher the symptom burden, the lower patients rate their quality of life. Not surprisingly, patients with CKD have rated symptom management as one of their top priorities,^4^ but sadly, CKD and dialysis patient symptoms are often under-recognized and undertreated. Because of the many unmet supportive care needs of this population, the International Society of Nephrology has recognized kidney supportive care as a vital part of integrated kidney care.^5^

In this issue of *Kidney360*, Cote *et al.* report that the health-related quality of life of patients recently discharged from the hospital after an episode of severe AKI is comparable with the known low quality of life of patients with advanced CKD.^6^ In a prospective observational study of 50 severe AKI survivors followed in a post-AKI clinic, the investigators found that patients' self-reported quality of life measured by the Treatment Burden Questionnaire (TBQ; scale 0–150, with higher scores indicating greater treatment burden) was not statistically different than that of a cohort of 50 patients with stage 4 and 5 CKD followed in a nephrology clinic in the same hospital. Despite being younger (median age 63 versus 72 years), having a lower Charlson Comorbidity Index score (5 versus 8), having a higher eGFR at 8–12 weeks of follow-up after discharge (60 versus 15 ml/min per m^2^), and taking fewer different medications daily (6 versus 11), the AKI survivors' median TBQ score did not differ from the patients with CKD (19.0 [6.5–43.8] versus 25 [12.8–43.0], *P* = 0.45). Most AKI survivors experienced stage 3 AKI (84%), required KRT (62%), and had an intensive care unit stay (64%). Their median length of hospital stay was 22 days. In the AKI survivors, the TBQ score was not associated with Kidney Disease Improving Global Outcomes AKI stage, recent exposure to kidney replacement treatment, or intensive care unit admission.

Cote *et al.* used the TBQ, a validated instrument to measure treatment burden. In patients with CKD and on dialysis, the TBQ score has a strong negative correlation with the health-related quality-of-life measure, Kidney Disease Quality of Life-36.^7^ Treatment burden is the work of being a patient and its effect on the patient's quality of life. The TBQ measures what patients do to take care of their health: adhering to complex treatment regimens, managing medications, changing lifestyle behaviors including diet and activity, visiting multiple health professionals, making and keeping appointments for physician office visits and laboratory and x-ray tests, addressing financial concerns and paperwork, performing self-monitoring, and maintaining relationships in which there may be difficulties with health care professionals.^6,8^

Previous studies have shed some light on the effect of AKI on quality of life. In the Veterans Affairs/National Institutes of Health Acute Renal Failure Trial Network study, 415 AKI survivors who received KRT during their hospitalization rated their Health Utilities Index score, a measure of health-related quality of life, at day 60 after enrollment (range 0–1 with 0 equivalent to death and 1 as perfect health). The Acute Renal Failure Trial Network study enrolled adults in critical care units who had AKI attributable to acute tubular necrosis plus sepsis or additional organ failure. The Health Utilities Index mean score was 0.40 and the modal score was 0; 27% reported a comprehensive health state that corresponded to a utility equal to or worse than death. As the authors noted, their finding of a very low post-AKI health-related quality-of-life score marked an urgent need to focus clinical management of AKI, not only on intensive care unit survival, but also on patient quality of life after discharge.^9^ In an American Association of Kidney Patients online anonymous survey of their members to learn about the patient experience of AKI, 124 responded. The respondents represented a convenience sample who had a high level of education compared with the average patient with kidney disease (27% had graduate or professional degrees and the majority were at least college graduates) and were disproportionately non-Hispanic White (77%). Yet, the findings were noteworthy; 84% of respondents reported that the AKI episode was very/extremely impactful on physical/emotional health and 57% reported being very/extremely concerned about AKI effects on work. The researchers identified qualitative themes including work-related concerns about declining performance and losing their job, family-related experiences of being rejected or misunderstood by their families and being a burden on them, and health care–related themes of overall lack of information and uncertainty about AKI and mismanagement and poor communication by health care professionals.^10^ Both studies called for further study of the effect of AKI on patients' quality of life using more comprehensive health-related quality-of-life measures.

In using the TBQ, Cote *et al.* added to the knowledge of the patient experience of AKI by identifying the treatment burden associated with AKI, including prolonged hospitalization. Because the TBQ score was not correlated with AKI risk factors, such as age, comorbidity score, and baseline eGFR, it is not clear from their study whether it was the AKI or the medical condition with which the AKI was associated, such as sepsis with hypotension in a patient with comorbidities, that resulted in the treatment burden. Additional limitations of the study are the small sample size with limited power to detect differences and the single site in which it was conducted.

Nonetheless, because the AKI survivors had treatment burden scores comparable with the patients with advanced CKD and have been previously known to have low health-related quality of life, this study signals the need to initiate the same strategies for kidney supportive care for both groups to improve their outcomes. Among other things, kidney supportive care is an approach to care that aims to improve the quality of life for people for whom kidney disease substantially affects their well-being. (The AKI survivors in the study by Cote *et al.* certainly had their well-being affected!) ^5^ Kidney supportive care is patient centered in that it seeks to determine what is most important to patients in treatment and uses shared decision making to ensure that patient values guide all decisions and that patients are coequals in determining their plan of care. In addition, kidney supportive care recognizes that individuals have physical, psychosocial, and spiritual needs. Not only should nephrology clinicians systematically screen patients for troublesome physical symptoms (using validated tools such as the Edmonton Symptom Assessment Scale-revised-Renal or the Integrated Palliative Outcome Scale-Renal) and treat them, but they should also involve the interdisciplinary care team to address patients' psychosocial and spiritual needs. Although the findings from the study by Cote *et al.* are preliminary, they alert the nephrology community to pay more attention to the treatment burden and quality of life experienced by AKI survivors.
